# Artificial Intelligence Versus Human-Controlled Doctor in Virtual Reality Simulation for Sepsis Team Training: Randomized Controlled Study

**DOI:** 10.2196/47748

**Published:** 2023-07-26

**Authors:** Sok Ying Liaw, Jian Zhi Tan, Khairul Dzakirin Bin Rusli, Rabindra Ratan, Wentao Zhou, Siriwan Lim, Tang Ching Lau, Betsy Seah, Wei Ling Chua

**Affiliations:** 1 Alice Lee Centre for Nursing Studies National University of Singapore Singapore Singapore; 2 Department of Media & Information Michigan State University East Lansing, MI United States; 3 Department of Medicine Yong Loo Lin School of Medicine National University of Singapore Singapore Singapore

**Keywords:** artificial intelligence, interprofessional education, interprofessional communication, sepsis care, team training, virtual reality, simulation, AI, health care education, nursing student, nursing education, medical education

## Abstract

**Background:**

Interprofessional communication is needed to enhance the early recognition and management of patients with sepsis. Preparing medical and nursing students using virtual reality simulation has been shown to be an effective learning approach for sepsis team training. However, its scalability is constrained by unequal cohort sizes between medical and nursing students. An artificial intelligence (AI) medical team member can be implemented in a virtual reality simulation to engage nursing students in sepsis team training.

**Objective:**

This study aimed to evaluate the effectiveness of an AI-powered doctor versus a human-controlled doctor in training nursing students for sepsis care and interprofessional communication.

**Methods:**

A randomized controlled trial study was conducted with 64 nursing students who were randomly assigned to undertake sepsis team training with an AI-powered doctor (AI-powered group) or with medical students using virtual reality simulation (human-controlled group). Participants from both groups were tested on their sepsis and communication performance through simulation-based assessments (posttest). Participants’ sepsis knowledge and self-efficacy in interprofessional communication were also evaluated before and after the study interventions.

**Results:**

A total of 32 nursing students from each group completed the simulation-based assessment, sepsis and communication knowledge test, and self-efficacy questionnaire. Compared with the baseline scores, both the AI-powered and human-controlled groups demonstrated significant improvements in communication knowledge (*P*=.001) and self-efficacy in interprofessional communication (*P*<.001) in posttest scores. For sepsis care knowledge, a significant improvement in sepsis care knowledge from the baseline was observed in the AI-powered group (*P*<.001) but not in the human-controlled group (*P*=.16). Although no significant differences were found in sepsis care performance between the groups (AI-powered group: mean 13.63, SD 4.23, vs human-controlled group: mean 12.75, SD 3.85, *P*=.39), the AI-powered group (mean 9.06, SD 1.78) had statistically significantly higher sepsis posttest knowledge scores (*P*=.009) than the human-controlled group (mean 7.75, SD 2.08). No significant differences were found in interprofessional communication performance between the 2 groups (AI-powered group: mean 29.34, SD 8.37, vs human-controlled group: mean 27.06, SD 5.69, *P*=.21). However, the human-controlled group (mean 69.6, SD 14.4) reported a significantly higher level of self-efficacy in interprofessional communication (*P*=.008) than the AI-powered group (mean 60.1, SD 13.3).

**Conclusions:**

Our study suggested that AI-powered doctors are not inferior to human-controlled virtual reality simulations with respect to sepsis care and interprofessional communication performance, which supports the viability of implementing AI-powered doctors to achieve scalability in sepsis team training. Our findings also suggested that future innovations should focus on the sociability of AI-powered doctors to enhance users’ interprofessional communication training. Perhaps in the nearer term, future studies should examine how to best blend AI-powered training with human-controlled virtual reality simulation to optimize clinical performance in sepsis care and interprofessional communication.

**Trial Registration:**

ClinicalTrials.gov NCT05953441; https://clinicaltrials.gov/study/NCT05953441

## Introduction

Delays in sepsis recognition and slow initiation of diagnostic work and treatment are associated with poor patient outcomes, including death [1-3]. It is thus necessary to ensure that health care professionals who have the first contact with patients with sepsis are trained to recognize and respond to sepsis in a time-critical manner [4]. Nurses, in particular, are often the first point of contact for assessing patients and are responsible for patient monitoring, so their abilities to recognize symptoms, escalate care, and initiate timely interventions for patients with or at risk of sepsis are of paramount importance [5,6]. However, internationally, it has been acknowledged that both nurses’ and nursing students’ knowledge of sepsis is often limited [7-9]. The importance of equipping nursing students entering the workforce with adequate knowledge and skills to assess patients, recognize symptoms, escalate care, and initiate initial management of patients with sepsis cannot be overemphasized because it is critical to reduce delays in the timely treatment of sepsis.

In addition to knowledge on sepsis recognition and management, nurses need to possess effective communication skills. Upon recognizing a patient with or at risk of sepsis, a nurse must be able to communicate patient concerns effectively to the medical team, for example, the junior doctors or attending doctors, as part of a process known as care escalation [10]. However, poor communication between nurses and doctors has been found to affect timely care escalation and review of patients, which can result in delayed treatment and contribute to patient harm and sentinel events [11-13]. One key reason that effective nurse-doctor communication remains a challenge is the lack of interprofessional learning experiences and interactions at the preregistration level [14,15]. This has prompted the incorporation of interprofessional education (IPE) in preregistration nursing and medical curriculums to prepare a collaborative practice-ready health workforce [14,15].

Although conventional simulation (ie, in-person simulation) has traditionally been a popular method for delivering team-based training in IPE, its implementation is often plagued by logistical issues, such as the availability of simulation facilities and facilitators, conflicting schedules among students from different health care professions and high costs involved [16]. This has resulted in the increased adoption of web-based virtual reality simulation (VRS), which can address the time and logistical constraints inherent in conventional simulation [17]. Studies, which evaluated on IPE delivered via VRS among preregistration health care students, have found improvements in attitudes toward collaboration, knowledge, and skills required for collaborative practice, interprofessional communication, and improved clinical behavior [18-20].

Specifically, a recent sepsis IPE program using VRS for undergraduate medical and nursing students has reported favorably on the use of VRS for interprofessional sepsis team training [10]. In the study, the medical and nursing students were required to log in simultaneously to the virtual platform to assume their avatar role for the sepsis team training. Significant improvements in sepsis knowledge and team communication skills for both the medical and nursing students were reported [10]. Furthermore, the sepsis IPE program fostered a greater understanding and appreciation of one another’s interprofessional roles in the care of patients with sepsis [10]. However, the authors pointed out 1 key limitation of human-controlled avatars: bringing together the medical and nursing students concurrently for frequent interprofessional team training was challenging because of scheduling conflicts. Similarly, Liaw et al [18] raised the concern that it would be unfeasible for all nursing students to form interprofessional teams with medical students to engage in doctor-nurse team training because the nursing student cohort tend to be disproportionately larger than the medical student cohort (eg, approximately 1500 vs 300 students). These 2 reasons motivated the development of medical doctor agents that are controlled by computer algorithms in VRS to allow nursing students to engage in interprofessional training.

Harnessing the power of artificial intelligence (AI)—a branch of computer science that builds intelligent computer systems capable of performing tasks that typically require human intelligence [21]—we developed an AI-powered doctor in our VRS for sepsis team training. Our pilot study showed positive evaluations of the acceptability, feasibility, and usability of the AI-powered VRS [22]. Having further worked on the expressiveness of the AI-powered doctor agent and intensified the dialogue training with learner-agent conversations, we aimed to evaluate the influence of the AI-powered doctor versus the human-controlled virtual doctor on nursing students’ sepsis care and interprofessional communication.

## Methods

### Study Design and Participants

We conducted a prospective 2-arm randomized controlled trial with a pretest-posttest study design. Social media platforms were used to recruit participants who were undertaking year 3 of their nursing courses in a local university. We used the rule of thumb for a pilot 2-arm trial sample size involving at least 55 participants [23]. Accounting for a 10% overall dropout rate, the total sample size was planned to be 64 participants. A total of 67 participants expressed their interest in the study. After screening for eligibility and obtaining written informed consent, the study coordinator randomized 65 participants using a web-based random number generator to either AI-powered or human-controlled groups [Multimedia Appendix 1].

### Ethics Approval

This study was approved by the National University of Singapore institutional review board (ref no. NUS-IRB-2022-202). Before the study intervention, both groups met the research team through a Zoom videoconference to receive information about the study and provide their written consent. They were assured that participation was entirely voluntary, and withdrawal would not affect their academic performance.

### Study Interventions

Participants in both the AI-powered and human-controlled groups were scheduled to participate in a 2-hour VRS on sepsis team training remotely. The participants in the AI-powered group were scheduled to undertake the VRS individually while being supported by the research team via Zoom chat for any enquiries. Participants in the human-controlled group were assigned to groups of 4 to 6 team players to engage in the VRS with medical students and facilitators. The design and development of both forms of VRS, which were grounded in experiential learning theory, has been described and evaluated in previous studies [18,22].

In both study interventions, participants were involved in 2 simulation scenarios. The first scenario simulated a morning medical round, involving a postoperative patient with early manifestations of sepsis, which required early goal-directed management of sepsis. The second scenario involved the same patient whose condition had deteriorated into septic shock and required airway management and fluid resuscitation. The participants were given time to read the case history before commencing each scenario. Both scenarios began with a nursing participant performing nursing assessment and management of the virtual patient using the ABCDE (Airway, Breathing, Circulation, Disability, Expose) approach, followed by communicating with an AI virtual doctor or a doctor avatar controlled by the medical student. A voice chatbot learning system was built using Google Cloud’s Dialogflow engine to train the AI doctor, which operates through deep neural networks to recognize and predict human-agent conversation patterns [21]. The AI doctor’s responses were modeled based on gathered conversational data set between nursing and medical students from previous studies [18,20].

**Figure 1:**
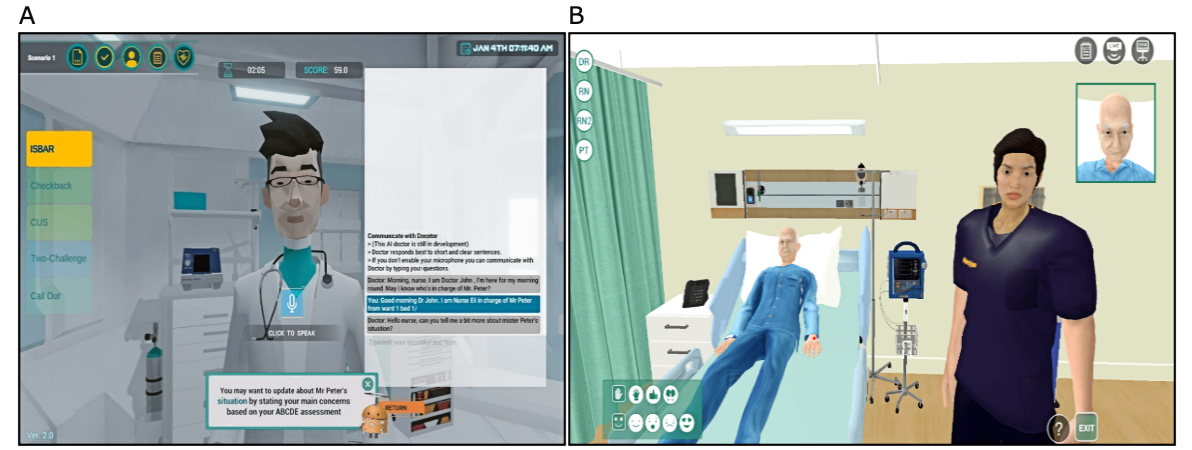
Viewpoints of different users. (A) Interacting with AI-powered doctor; (B) interacting with human-controlled doctor avatar.

Figure 1 illustrates the participants’ viewpoints when interacting with the AI-powered doctor or the medical-student-controlled avatar. We adapted communication strategies from the TeamSTEPPS (Team Strategies and Tools to Enhance Performance and Patient Safety) curriculum, which included ISBAR (Identity, Situation, Background, Assessment and Recommendation) and CUS (Concerned, Uncomfortable and Safety) feedback to acknowledge, call out, and check back [24]. In these scenarios, the nursing participants were expected to use communication strategies to communicate the assessment findings to the AI-powered doctor or human-controlled doctor avatar.

Each simulation scenario lasted 15 to 20 minutes and was followed by a debriefing to enable participants to receive feedback on their performance regarding the assessment and management of sepsis and septic shock and their interprofessional communication. As illustrated in Figure 1, the AI-powered group received a self-directed debrief checklist to review their performance, whereas the human-controlled group engaged in facilitator-led debriefing. In both group debriefings, the ABCDE and TeamSTEPPS communication tools provided the frameworks to guide learning through feedback.

### Data Collection and Instrument

We administered the knowledge tests and self-efficacy scale before (baseline) and immediately after (posttest) interventions for both groups. The 8-item communication knowledge and 18-item sepsis knowledge tests were developed and content validated by a multidisciplinary team comprising a medical doctor, an advanced practice nurse, and nursing academics. This study reported a high Cronbach α of .81. The Patient Clinical Information Exchange and Interprofessional Communication Self-Efficacy Scale, a 6-item questionnaire using a 0-100 Likert scale developed by Hernández-Padilla et al [25], was used to measure participants’ perceptions of self-efficacy in team communication based on the ISBAR communication strategy. A high internal consistency with a Cronbach α of .93 was obtained for the Patient Clinical Information Exchange and Interprofessional Communication Self-Efficacy Scale in this study.

Participants from both groups were scheduled to undertake simulation-based assessment within 2 weeks of postintervention to determine their sepsis care and interprofessional communication performance. Participants were given a case history to read and an orientation of the simulation room with a manikin setup. The simulation-based assessment involved the participants performing the nursing assessment and management of the manikin, which displayed signs and symptoms of deterioration, and communicating with the doctor to provide team care. Each simulation assessment lasted approximately 15 minutes, and the entire process was recorded. The recorded videos were sent for rating by 2 assessors, who were blinded to the groupings. The assessors rated the communication and sepsis care performances independently using a validated team communication scale and the RAPIDS (Rescuing A Patient In Deteriorating Situation) tool. The team communication scale comprising a 9-item checklist was developed by the research team based on observable nurse-doctor communication using the TeamSTEPPS communication strategies. The scale was content validated by an interprofessional team of 5 nursing academics and clinicians. The RAPIDS tool was adopted from a previous study to measure nurses’ simulation performance in assessing and managing a deteriorating patient [26]. Interrater reliability across the 2 assessors was computed based on their independent scoring on the video-recorded performances using the validated team communication scale and RAPIDS tool. An overall κ value of 0.832 was reported, indicating good interrater agreement.

### Data Analysis

We applied descriptive statistics, chi-square tests and *t* tests, to analyze the demographic characteristics of the study population. We computed the paired sample *t* test to examine significant changes between the baseline and posttest performance scores and used ANOVA to determine differences in the posttest scores between the groups. The level of statistical significance was set at *P*<.05.

## Results

### Demographic Characteristics

Sixty-four nursing students were recruited in the study. The majority were women (50/64, 78%) and Chinese (51/64, 80%) and had a mean age of 22.2 (SD 2.20) years. No significant differences were observed in the baseline characteristics, including age (*P*=.49), gender (*P*=.55), and ethnicity (*P*=.54) between the AI doctor and human doctor groups (see Table 1). This supported the homogeneity of participants between the 2 groups.

**Table 1:** Demographic characteristics (N=64).

Characteristic			Overall		AI^a^ doctor group (n=32)		Human doctor group (n=32)		*P* value	
Age (years), mean (SD)			22.2 (2.20)		22.0 (1.83)		22.4 (2.52)		.49	
**Gender, n (%)**										.55
	Man	14 (22)		6 (19)		8 (25)				
	Woman	50 (78)		26 (81)		24 (75)				
**Ethnicity, n (%)**										.54
	Chinese	51 (80)		25 (78)		26 (81)				
	Indian	4 (6)		2 (6)		2 (6)				
	Malay	2 (3)		2 (6)		0 (0)				
	Other	7 (11)		3 (9)		4 (13)				

^a^AI: artificial intelligence.

### Sepsis Care Knowledge and Performance

The simulation-based assessment revealed no significant differences in the sepsis care performance scores between the AI-powered and human-controlled groups (*F*_1,62_=0.75, *P*=.39, η^2^=0.012), though a higher mean score was observed in the AI-powered group (see Table 2). In contrast, significant differences were noted in the sepsis care knowledge scores between the participants in the AI-powered and human-controlled groups (*F*_1,62_=7.37, *P*=.009, η^2^=0.106). As shown in Table 2, the AI-powered group reported higher sepsis care knowledge mean scores (mean 9.06, SD 1.78) than the human-controlled group (mean 7.75, SD 2.08). Within-group comparison demonstrated a significant increase in sepsis care knowledge from the baseline in the AI-powered group (t_31_=−5.21, *P*<.001) but not the human-controlled (t_31_=−1.43, *P*=.16) group.

**Table 2:** Comparison of study outcomes within and between groups (N=64).

Test			AI^a^ group (n=32)									Human group (n=32)									Between group, *F* value (*df*)			*P* value
			Pretest, mean (SD)		Posttest, mean (SD)		Within group, *t* value (*df*)		*P* value		Pretest, mean (SD)			Posttest, mean (SD)		Within group, *t* value (*df*)		*P* value						
**Sepsis care**																								
	Knowledge	6.91 (1.63)		9.06 (1.78)		−5.21 (31)		<.001		7.03 (2.25)			7.75 (2.08)		−1.43 (31)		.16		7.37 (1,62)			.009		
	Performance	—^b^		13.63 (4.23)		—		—		—			12.75 (3.85)		—		—		0.75 (1,62)			.39		
**Communication**																								
	Self-efficacy	53.3 (10.4)		60.1 (13.3)		−3.78 (31)		<.001		59.9 (16.4)			69.6 (14.4)		−4.52 (31)		<.001		7.50 (1,62)			.008		
	Knowledge	2.47 (1.48)		3.88 (1.48)		−5.23 (31)		<.001		2.75 (1.44)			4.19 (1.97)		−3.60 (31)		.001		0.514 (1,62)			.48		
	Performance	—		29.34 (8.37)		—		—		—			27.06 (5.69)		—		—		1.62 (1,62)			.21		

^a^AI: artificial intelligence.

^b^Not applicable.

### Communication Knowledge, Performance, and Self-efficacy

As presented in Table 2, within-group comparison revealed significant improvements in communication knowledge and self-efficacy from baseline levels in both AI-powered and human-controlled groups. Although between-groups comparison showed no significant differences in communication knowledge scores (*F*_1,62_=0.514, *P*=.48, η^2^=0.008) and communication performance scores (*F*_1,62_=1.62, *P*=.21, η^2^=0.026), the human-controlled group reported higher communication self-efficacy mean scores (mean 69.6, SD 14.4) than the AI-powered group (mean 60.1, SD 13.3).

## Discussion

### Principal Findings

To the best of our knowledge, this is one of the first studies to evaluate the effectiveness of AI-powered VRS by comparing it with human-controlled VRS. This randomized controlled trial study did not demonstrate any significant differences in sepsis performance and interprofessional communication performance between participants in the AI-powered and human-controlled groups. However, we found significant differences in sepsis care knowledge and communication self-efficacy between the 2 groups. Although the AI-powered group obtained significantly higher sepsis care knowledge scores than the human-controlled group, the human-controlled group reported significantly higher self-efficacy in interprofessional communication than in the AI-powered group. The underlying learning principles and theories that guided the use of AI and human-controlled VRS could be applied to explain these findings.

The simulation-based assessment performance outcomes did not reveal inferiority of AI-powered VRS regarding either sepsis care or communication skills performance when compared with human-controlled VRS. Despite the different medical team virtual player agents, both VRS approaches offered experiential learning and cognitive tools that allowed the nursing students to practice the assessment and management of sepsis and team communication skills through role playing and reflection. An earlier study demonstrated the need for both cognitive tools and experiential learning modalities to support the development of a shared mental model and optimal teamwork delivery [27]. The application of experiential learning using various simulation modalities, including computer-based simulation and manikin-based simulation, has demonstrated positive outcomes in clinical performance and team communication related to the care of patients with clinical deterioration [28,29]. Besides grounding the study in experiential learning theory, we also ensured close alignment between the simulation task and the clinical task in both VRS groups. Thus, findings from this study provided further evidence to support Hamstra et al’s [30] recommendation of focusing on high functional fidelity rather than physical fidelity of simulation to develop desired performance outcomes.

Interestingly, our findings demonstrated that the AI-powered group had significantly higher sepsis knowledge scores than the human-controlled group. This finding supported our earlier qualitative data in which the AI-powered doctor was perceived by the nursing students as a more knowledgeable agent than the doctor agent controlled by medical students [22]. Although both AI- and human-controlled VRS approaches were based on experiential learning, they used different approaches to support experiential learning. Kiili’s [31] experiential gaming model was applied to build the game design of AI-powered VRS, which involved multiple quizzes as a form of challenge for learners to interpret and problem-solve patient assessment data with an awarded point system. The AI-powered virtual agent was also designed to involve learners in a reasoning process through questioning. The importance of using the AI-powered virtual agent to facilitate knowledge construction, rather than to provide knowledge, has been highlighted in several studies; this approach ensures the development of learners’ reasoning process for knowledge construction [32,33]. Using a self-regulated learning approach, feedback was delivered through a checklist format with evidence-based decision-making rationales. With growing attention given to the application of AI to support assessment, AI-driven learning analytics for assessment can be embedded into the VRS to generate feedback to students, as well as to provide informative data for educators to track learners’ learning outcomes [34].

Although no significant difference was noted regarding interprofessional communication performance, the human-controlled group reported a significantly higher level of self-efficacy for interprofessional communication than in the AI-powered group. Unlike AI-powered groups, participants in the human-controlled group were given the opportunity to engage in social interactions among learners through role playing and debriefing. The theory of social constructivism has emphasized the importance of learning from social interaction [35], which has been predominantly applied in multiuser virtual worlds to underpin learning activities [36]. Thus, social constructivism could be applied to explain how our human-controlled multiuser VRS might bring about higher self-efficacy of interprofessional communication than in the AI-powered simulation. Although we have been working toward improving the AI-powered doctor’s affective states consisting of facial expressions and comprehension of natural conversation, we acknowledged that the fidelity and authenticity of our AI-powered agent poses challenges in promoting social interaction through the human-AI conversation. This suggested the need for further innovative development to drive a more sociably enabled AI using emotion-expressive virtual agents, which can be realized by seeking progression in multimodal computing and machine learning [37,38].

The lack of substantial differences in performance between AI and human-controlled VRS highlighted the potential role of AI-powered VRS in supporting nursing students with interprofessional training, particularly in circumstances when the accessibility and availability of medical students are lacking. In addition, variations in learning outcomes between the groups suggested the benefits of combining the different simulation modalities to provide an optimal learning approach. Our previous study recommended the use of scaffolding for the instructional sequence of interprofessional learning activities within blended learning environments [39]. Thus, a blended learning approach commencing with concept building using AI-powered VRS, followed by experiential learning with medical students in a virtual environment, and subsequently using face-to-face simulation-based interprofessional learning could be implemented and evaluated in future studies.

### Limitations

The limitations that warrant attention are few. First, we acknowledged the variations in experiential learning approach between the 2 simulation modalities as confounding variables. However, the performance outcome measurements used in this study were closely aligned with the learning objectives. Second, despite evaluating participants’ performance using simulation-based assessment and a validated tool, this was limited by an immediate posttest performance in the simulation setting. Third, similar to the performance test, we did not measure the long-term retention of knowledge and level of self-efficacy. Thus, future studies could evaluate learning outcomes over a longer period and measure the impact in the clinical setting. Finally, the effectiveness of AI-powered VRS was not optimized by allowing participants to have more than one exposure as the intent was to enable deliberate practice opportunities.

### Conclusions

An AI virtual doctor agent was embedded in a virtual environment to engage interprofessional team training of nursing students on sepsis care. The performance outcomes from simulation-based assessment did not suggest that the AI-powered VRS provided inferior sepsis care and interprofessional communication training when compared with a human-controlled VRS. This shed light on the effectiveness of AI-powered medical team players in supporting nursing students with interprofessional learning where the opportunity to form interprofessional teams with medical students is lacking. Our findings suggested the need for further innovative development in AI-powered VRS to promote social connectedness with learners and support AI-driven learning analytics for assessment. Given the varied learning outcomes between AI-powered and human-controlled VRS approaches, our study recommended blending them along with face-to-face simulation to optimize students’ performance in sepsis care and interprofessional communication.

## Appendix

Multimedia Appendix 1 CONSORT Flow Diagram.

Multimedia Appendix 2 CONSORT-EHEALTH (Consolidated Standards of Reporting Trials of Electronic and Mobile Health Applications and Online Telehealth; V1.6.1) checklist.

## Abbreviations

ABCDE: Airway, breathing, circulation, disability, exposure
AI: artificial intelligence
CUS: Concerned, Uncomfortable, and Safety
IPE: interprofessional education
ISBAR: Identity, Situation, Background, Assessment, and Recommendation
RAPIDS: rescuing a patient in deteriorating situation
TeamSTEPPS: team strategies and tools to enhance performance and patient safety
VRS: virtual reality simulation

## Data Availability

The data sets collected or analyzed during the study are available from the corresponding author on reasonable request.

## References

[1] Levy MM, Rhodes A, Phillips GS, Townsend SR, Schorr CA, Beale R, Osborn T, Lemeshow S, Chiche JD, Artigas A, Dellinger RP (2015). Surviving sepsis campaign: association between performance metrics and outcomes in a 7.5-year study. Crit Care Med.

[2] Neilson HK, Fortier JH, Finestone PJ, Ogilby CM, Liu R, Bridges EJ, Garber GE (2023). Diagnostic delays in sepsis: lessons learned from a retrospective study of Canadian medico-legal claims. Crit Care Explor.

[3] Rhee C, Jones TM, Hamad Y, Pande A, Varon J, O'Brien C, Anderson DJ, Warren DK, Dantes RB, Epstein L, Klompas M, Centers for Disease Control and Prevention (CDC) Prevention Epicenters Program (2019). Prevalence, underlying causes, and preventability of sepsis-associated mortality in US acute care hospitals. JAMA Netw Open.

[4] Schlapbach LJ, Kissoon N, Alhawsawi A, Aljuaid MH, Daniels R, Gorordo-Delsol LA, Machado F, Malik I, Nsutebu EF, Finfer S, Reinhart K (2020). World sepsis day: a global agenda to target a leading cause of morbidity and mortality. Am J Physiol Lung Cell Mol Physiol.

[5] Chua WL, Teh CS, Basri MABA, Ong ST, Phang NQQ, Goh EL (2023). Nurses' knowledge and confidence in recognizing and managing patients with sepsis: a multi-site cross-sectional study. J Adv Nurs.

[6] Harley A, Johnston ANB, Denny KJ, Keijzers G, Crilly J, Massey D (2019). Emergency nurses' knowledge and understanding of their role in recognising and responding to patients with sepsis: a qualitative study. Int Emerg Nurs.

[7] Harley A, Massey D, Ullman AJ, Reid-Searl K, Schlapbach LJ, Takashima M, Venkatesh B, Datta R, Johnston ANB (2021). Final year nursing student's exposure to education and knowledge about sepsis: a multi-university study. Nurse Educ Today.

[8] Nucera G, Esposito A, Tagliani N, Baticos CJ, Marino P (2018). Physicians' and nurses' knowledge and attitudes in management of sepsis: an Italian study. J Health Soc Sci.

[9] Storozuk SA, MacLeod MLP, Freeman S, Banner D (2019). A survey of sepsis knowledge among Canadian emergency department registered nurses. Australas Emerg Care.

[10] Chua WL, Ooi SL, Chan GWH, Lau TC, Liaw SY (2022). The effect of a sepsis interprofessional education using virtual patient telesimulation on sepsis team care in clinical practice: mixed methods study. J Med Internet Res.

[11] Chua WL, Legido-Quigley H, Jones D, Hassan NB, Tee A, Liaw SY (2020). A call for better doctor-nurse collaboration: a qualitative study of the experiences of junior doctors and nurses in escalating care for deteriorating ward patients. Aust Crit Care.

[12] Ede J, Petrinic T, Westgate V, Darbyshire J, Endacott R, Watkinson PJ (2021). Human factors in escalating acute ward care: a qualitative evidence synthesis. BMJ Open Qual.

[13] O'Neill SM, Clyne B, Bell M, Casey A, Leen B, Smith SM, Ryan M, O'Neill M (2021). Why do healthcare professionals fail to escalate as per the early warning system (EWS) protocol? A qualitative evidence synthesis of the barriers and facilitators of escalation. BMC Emerg Med.

[14] Au S (2023). The outcomes of interprofessional education in prelicensure nursing education: an integrative review. Nurse Educ Today.

[15] Nelson S, White CF, Hodges BD, Tassone M (2017). Interprofessional team training at the prelicensure level: a review of the literature. Acad Med.

[16] Liaw SY, Zhou WT, Lau TC, Siau C, Chan SWC (2014). An interprofessional communication training using simulation to enhance safe care for a deteriorating patient. Nurse Educ Today.

[17] Almousa O, Zhang R, Dimma M, Yao J, Allen A, Chen L, Heidari P, Qayumi K (2021). Virtual reality technology and remote digital application for tele-simulation and global medical education: an innovative hybrid system for clinical training. Simul Gaming.

[18] Liaw SY, Ooi SW, Rusli KDB, Lau TC, Tam WWS, Chua WL (2020). Nurse-physician communication team training in virtual reality versus live simulations: randomized controlled trial on team communication and teamwork attitudes. J Med Internet Res.

[19] Liaw SY, Soh SLH, Tan KK, Wu LT, Yap J, Chow YL, Lau TC, Lim WS, Tan SC, Choo H, Wong LL, Lim SM, Ignacio J, Wong LF (2019). Design and evaluation of a 3D virtual environment for collaborative learning in interprofessional team care delivery. Nurse Educ Today.

[20] Liaw SY, Choo T, Wu LT, Lim WS, Choo H, Lim SM, Ringsted C, Wong LF, Ooi SL, Lau TC (2021). Wow, woo, win"- healthcare students' and facilitators' experiences of interprofessional simulation in three-dimensional virtual world: a qualitative evaluation study. Nurse Educ Today.

[21] Chan KS, Zary N (2019). Applications and challenges of implementing artificial intelligence in medical education: integrative review. JMIR Med Educ.

[22] Liaw SY, Tan JZ, Lim S, Zhou W, Yap J, Ratan R, Ooi SL, Wong SJ, Seah B, Chua WL (2023). Artificial intelligence in virtual reality simulation for interprofessional communication training: mixed method study. Nurse Educ Today.

[23] Sim J, Lewis M (2012). The size of a pilot study for a clinical trial should be calculated in relation to considerations of precision and efficiency. J Clin Epidemiol.

[24] Ross JG, Latz E, Meakim CH, Mariani B (2021). TeamSTEPPS curricular-wide integration: baccalaureate nursing students' knowledge, attitudes, and perceptions. Nurse Educ.

[25] Hernández-Padilla JM, Cortés-Rodríguez AE, Granero-Molina J, Fernández-Sola C, Correa-Casado M, Fernández-Medina IM, López-Rodríguez MM (2019). Design and psychometric evaluation of the 'clinical communication self-efficacy toolkit'. Int J Environ Res Public Health.

[26] Liaw SY, Scherpbier A, Klainin-Yobas P, Rethans JJ (2011). Rescuing A Patient In Deteriorating Situations (RAPIDS): an evaluation tool for assessing simulation performance on clinical deterioration. Resuscitation.

[27] Liaw SY, Wu LT, Wong LF, Soh SLH, Chow YL, Ringsted C, Lau TC, Lim WS (2019). "Getting everyone on the same page": interprofessional team training to develop shared mental models on interprofessional rounds. J Gen Intern Med.

[28] Ashokka B, Dong C, Law LSC, Liaw SY, Chen FG, Samarasekera DD (2020). A BEME systematic review of teaching interventions to equip medical students and residents in early recognition and prompt escalation of acute clinical deteriorations: BEME guide no. 62. Med Teach.

[29] Liaw SY, Chan SWC, Chen FG, Hooi SC, Siau C (2014). Comparison of virtual patient simulation with mannequin-based simulation for improving clinical performances in assessing and managing clinical deterioration: randomized controlled trial. J Med Internet Res.

[30] Hamstra SJ, Brydges R, Hatala R, Zendejas B, Cook DA (2014). Reconsidering fidelity in simulation-based training. Acad Med.

[31] Kiili K (2005). Content creation challenges and flow experience in educational games: the IT-emperor case. Internet High Educ.

[32] Graesser A, McNamara D (2010). Self-regulated learning in learning environments with pedagogical agents that interact in natural language. Educ Psychol.

[33] Matsuda N, Weng W, Wall N (2020). The effect of metacognitive scaffolding for learning by teaching a teachable agent. Int J Artif Intell Educ.

[34] Dai CP, Ke F (2022). Educational applications of artificial intelligence in simulation-based learning: a systematic mapping review. Comput Educ Artif Intell.

[35] Piaget J, Mussen PH (1970). Piaget's theory. Carmichael's Manual of Child Psychology. 3rd Edition.

[36] Liaw SY, Carpio GAC, Lau Y, Tan SC, Lim WS, Goh PS (2018). Multiuser virtual worlds in healthcare education: a systematic review. Nurse Educ Today.

[37] Guo YR, Goh DHL (2015). Affect in embodied pedagogical agents: meta-analytic review. J Educ Comput Res.

[38] Lawson AP, Mayer RE, Adamo-Villani N, Benes B, Lei X, Cheng J (2021). Do learners recognize and relate to the emotions displayed by virtual instructors?. Int J Artif Intell Educ.

[39] Liaw SY, Tan KK, Wu LT, Tan SC, Choo H, Yap J, Lim SM, Wong L, Ignacio J (2019). Finding the right blend of technologically enhanced learning environments: randomized controlled study of the effect of instructional sequences on interprofessional learning. J Med Internet Res.

