# Supplementary figures and images for: Artificial Intelligence Versus Human-Controlled Doctor in Virtual Reality Simulation for Sepsis Team Training: Randomized Controlled Study
Source: J Med Internet Res. 2023 Jul 26;25:e47748. doi: 10.2196/47748 (PMC10413090; doi:10.2196/47748)

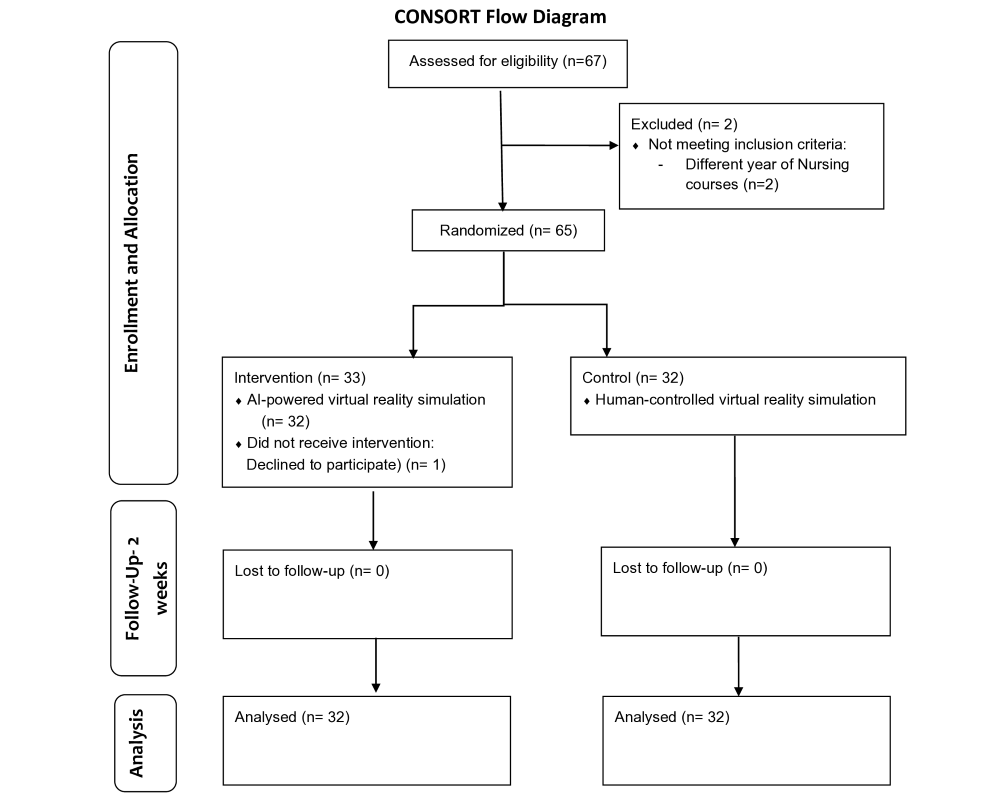

Supplement: Multimedia Appendix 1 [file jmir_v25i1e47748_app1.png]
